# Endoscopically guided interventional photodynamic therapy for orthotopic pancreatic ductal adenocarcinoma based on NIR-II fluorescent nanoparticles

**DOI:** 10.7150/thno.84164

**Published:** 2023-08-06

**Authors:** Kang Chen, Baoli Yin, Quanneng Luo, Yi Liu, Yi Wang, Yan Liao, Yuhang Li, Xu Chen, Bo Sun, Ning Zhou, Hongwen Liu, Chuang Peng, Sulai Liu, Wei Cheng, Guosheng Song

**Affiliations:** 1 Department of Hepatobiliary Surgery, Hunan Provincial People's Hospital (The First Affiliated Hospital of Hunan Normal University), Changsha 410005, P. R. China.; 2 State Key Laboratory of Chemo/Bio-Sensing and Chemometrics, College of Chemistry and Chemical Engineering, Hunan University, Changsha 410082, P. R. China.; 3 Xiangyue Hospital Affiliated to Hunan Institute of Parasitic Diseases, National Clinical Center for Schistosomiasis Treatment, Yueyang 414000, P. R. China.; 4 Translational Medicine Laboratory of Pancreas Disease of Hunan Normal University, Changsha 410005, P. R. China.; 5 Central Laboratory of Hunan Provincial People's Hospital (The First Affiliated Hospital of Hunan Normal University), Changsha 410005, P. R. China.

**Keywords:** Endoscopy, NIR-II, Photodynamic therapy, Pancreatic cancer

## Abstract

**Rationale:** Pancreatic cancer, comprising mostly pancreatic ductal adenocarcinoma (PDAC), is a highly malignant disease, typically known as a hypoxic tumor microenvironment. The application of PDT in pancreatic cancer in clinic is still hampered by several shortcomings, including the (i) deep location of pancreatic cancer, (ii) tissue damage induced by optical fibers, (iii) hypoxic microenvironment, (iv) short excitation wavelengths of traditional photosensitizers, and (v) poor delivery efficiency of photosensitizers.

**Methods:** We designed an organic nanoparticle as photosensitizer for near-infrared II (NIR-II) fluorescent (FL) imaging that exerts a type I PDT effect on deep orthotopic pancreatic tumors under excitation by a NIR (808 nm) laser.

**Results:** This novel photosensitizer exhibits enhanced accumulation in orthotopic pancreatic cancer in mice and could be used to effectively detect pancreatic cancer and guide subsequent laser irradiation for accurate PDT of deep pancreatic cancer. In addition, we built an endoscopic platform monitored by NIR-II FL imaging to achieve minimally invasive endoscopically guided interventional photodynamic therapy (EG-iPDT) with efficient inhibition of orthotopic pancreatic cancer, which prolonged overall survival up to 78 days compared to PBS + EG-iPDT group (*p < 0.05) in a mouse model.

**Conclusions:** Minimally invasive EG-iPDT has promise as an intraoperative treatment for early-stage or unresectable or metastatic pancreatic cancer.

## Introduction

In 2020, there were more than 490,000 new cases of pancreatic cancer and 460,000 deaths related to the disease worldwide 1, 2. The 5-year survival rate of pancreatic cancer is less than 10%, and it is projected to become the second leading cause of cancer-related death globally by 2030^3^. Most pancreatic tumors are found in the late stage, and treatment mainly involves adjuvant chemotherapy and surgery depending on the extent of vascular infiltration and the presence of distant metastases 4. For microscopic lesions that appear early in pancreatic cancer, surgery is usually the first line of treatment. However, traditional surgical treatment is very traumatic, and patients recover slowly. Several characteristics of the disease, such as the dense stroma, hypoxic tumor microenvironment (TME) and abnormal blood vessel perfusion, obstruct the intratumorally delivery of drugs from blood circulation to the targeted region, which constitutes a massive obstacle to therapeutic efficacy 5.

Recently, photodynamic therapy (PDT) has attracted increasing attention as a promising clinical method for treating cancer, such as cholangiocarcinoma and oral cancer due to its low systemic toxicity and minimal invasiveness 6, 7. At present, the photosensitizers used in PDT for pancreatic cancer in clinic mainly included Ce6 (excited by a 400 nm laser) 8, mTHPC (excited by a 652 nm laser) 9 and verteporfin (excited by a 689 nm laser) 10. However, the short excitation wavelengths limited the laser penetration depth for treating deep-seated pancreatic cancer. Moreover, the desmoplastic stroma in the pancreatic TME hinders the delivery of photosensitizers from the circulation into the tumor. Since the efficacy of typical type II PDT is highly dependent on oxygen concentration, the hypoxia as an important characteristic of pancreatic cancer has been speculated to be a key driver of resistance to PDT 11. In addition, the main PDT modalities for patients with pancreatic cancer are currently percutaneous/transgastric/duodenal ablation under the guidance of ultrasound (US), CT or endoscopic ultrasonography (EUS)^8^, ^9^, ^12^, which may lead to gastrointestinal hemorrhage, duodenal obstruction and vascular damage or incomplete ablation, potentially resulting in treatment failure and tumor recurrence^13^. Thus, the application of PDT in orthotopic pancreatic cancer is mainly limited by (i) insufficient laser penetration, (ii) insufficient photosensitizer delivery, (iii) tumor-oxygenation dependency and (iv) intervention-related complications. Radiofrequency ablation is a method of killing tumors through high temperature, which is currently widely used in small liver cancer, but has been less reported in pancreatic tumors. Combining photodynamic therapy with photothermal therapy has the potential to effectively eliminate tumors.

Given the three key problems of conventional photosensitizers being excited by short wavelengths, the special challenge presented by the hypoxic TME in pancreatic cancer, and the invasive injury caused by traditional PDT, we adopted a strategy of combining a minimally invasive endoscopically guided method with a multifunctional photosensitizer for NIR-II FL based type I PDT for orthotopic pancreatic cancer. First, we designed a large conjugated system (A-D-A'-D-A) by introducing two electron-absorbing groups at both ends of the D-A'-D core. Due to the introduction of two electron-absorbing groups, the absorption wavelength was significantly redshifted, and the FL emission was redshifted to the NIR-II region by expansion of the conjugated system. Next, we prepared a theranostic agent via the nanoprecipitation method as photosensitizers that could be excited at long wavelengths (~800 nm) and generate **•**OH by the type I PDT process. The BTz-IC NPs exhibited a long-excitation wavelength, satisfactory NIR-II FL intensity, efficient **•**OH production capability and stable photostability *in vitro*. This type I PDT system was able to overcome the hypoxic property of the pancreatic cancer TME and enhance the photodynamic effect.

Endoscopy, as a minimally invasive surgical adjunct can reduce trauma while enabling clearer and more intuitive detection of tumors, including deeper microscopic tumors that are difficult to detect with the naked eye. The advantages of endoscopy applied in hepato-pancreato-biliary surgery included faster recovery, a shorter hospital stay, decreased postoperative pain and a reduced risk of infectious complications 14. To overcome the above problems, we hypothesized that endoscopically guided interventional PDT (EG-iPDT) combined with NIR-II nanoagents could improve the efficacy of therapy for orthotopic pancreatic cancer. Herein, we built a small-animal endoscopic platform to explore EG-iPDT for early-stage orthotopic pancreatic tumors in mice by combining a novel photosensitizer with a long excitation wavelength (808 nm) and NIR-II FL imaging. After intravenous injection, the BTz-IC NPs selectively accumulated at subcutaneous/orthotopic pancreatic tumors, as confirmed by NIR-II FL imaging, and exerted an effective outcome via the type I photodynamic effect *in vivo*. After EG-iPDT, the orthotopic pancreatic tumors were significantly suppressed, and the overall survival was extended up to 78 days. Thus, the combination of interventional technology and PDT enabled the precise and efficient treatment of deep pancreatic tumors.

## Results

### Design, synthesis, and characterization of the BTz-IC NPs

**Scheme 1:**
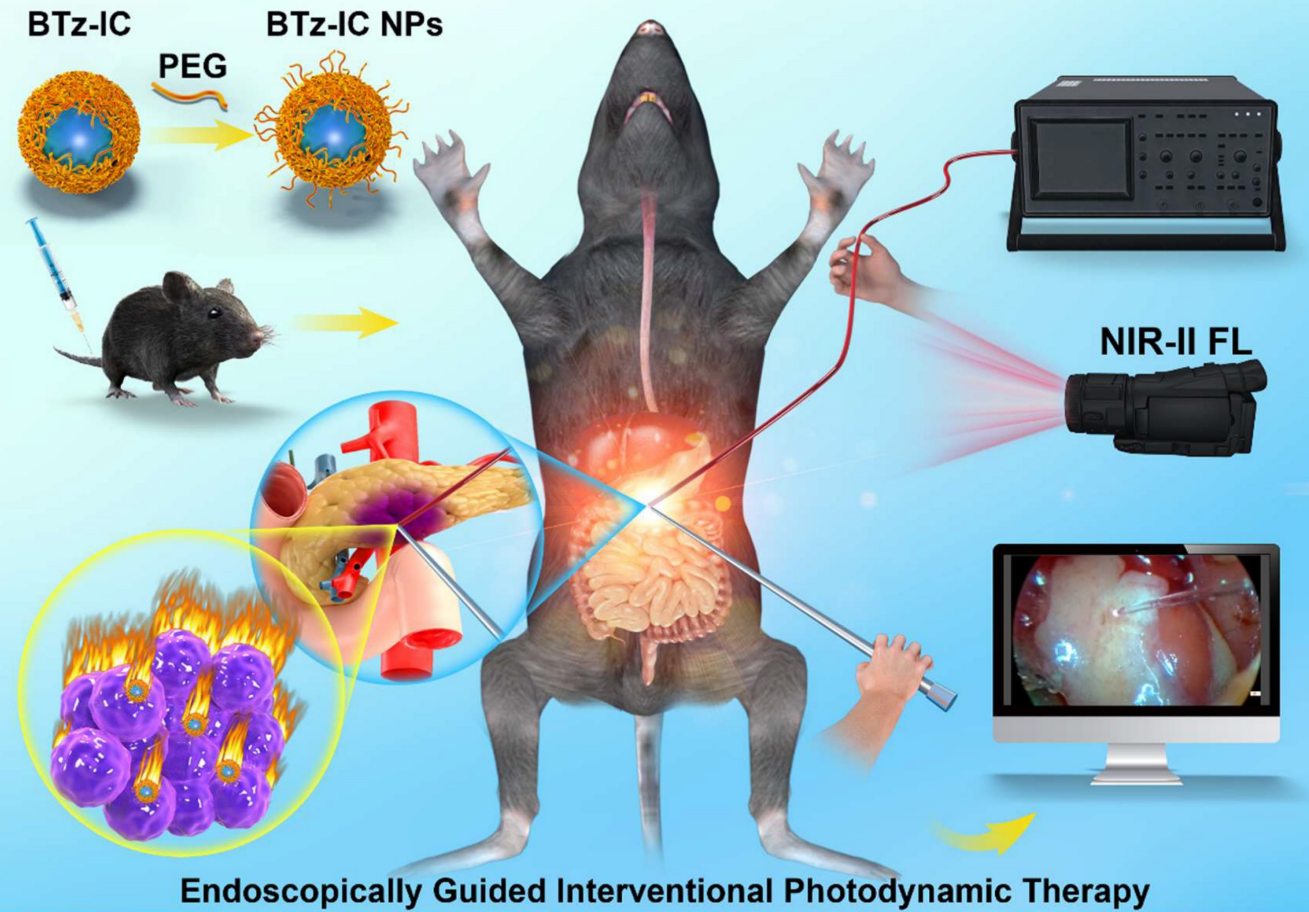
Schematic illustration shows the design of NIR-II theranostic agents for Endoscopically Guided Interventional Photodynamic Therapy (EG-iPDT).

**Figure 1:**
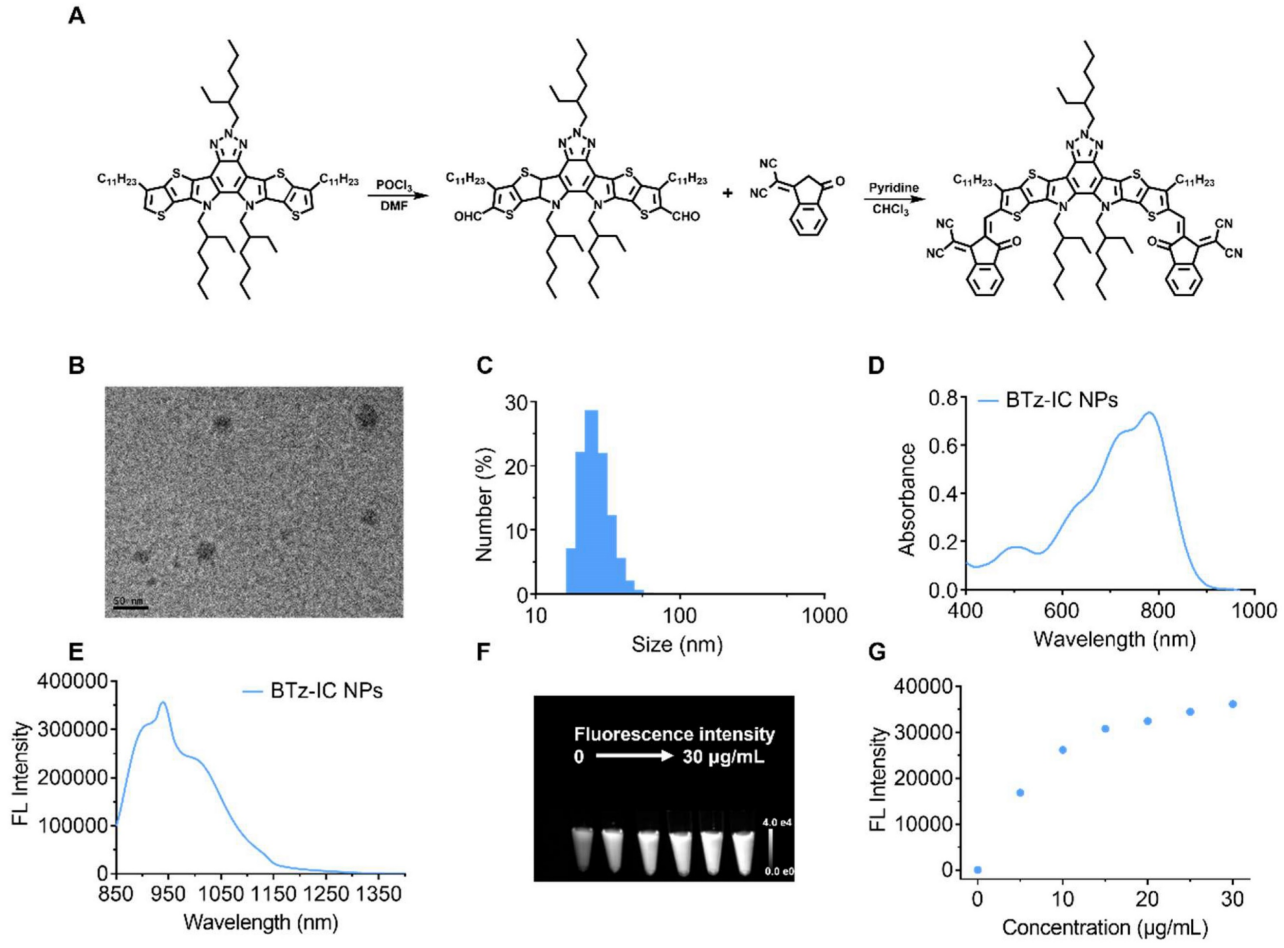
Synthesis and characterization of the BTz-IC NPs. (A) Synthetic routes for the BTz-IC NPs. (B) Transmission electron microscopy (TEM) (Scale bar = 50 nm). (C) The hydrodynamic size distribution of the BTz-IC NPs. (D) UV-vis-NIR absorption spectra of the BTz-IC NPs. (E) The FL emission curve of the BTz-IC NPs (power density, 1.0 W/cm^2^). (F) *In vitro* NIR-II FL (filter, 1,040 nm) images of the BTz-IC NPs at different concentration, and (G) the FL intensity of the BTz-IC NPs in aqueous solutions at different concentrations (0, 5, 10, 15, 20, 25 and 30 μg/mL).

First, we synthesized a NIR-II molecule by a straightforward two-step reaction to produce a conjugated system (A-D-A'-D-A), named BTz-IC. To create water-soluble NIR-II NPs, the surfactant DSPE-mPEG was coated onto BTz-IC by nanocoprecipitation (Scheme 1 and Figure 1A). Transmission electron microscopy (TEM) (Figure 1B) showed that the BTz-IC NPs were spherical and uniform in shape with an average diameter of ~18.4 nm. The BTz-IC NPs were water soluble, with a size of approximately 24.3 nm, as determined by dynamic light scattering (DLS) (Figure 1C), which is consistent with the TEM results, and a zeta potential of approximately -31.6 mV (Figure S1). Proton nuclear magnetic resonance (^1^H NMR) confirmed the successful synthesis of the BTz-IC (Figure S2). The obtained BTz-IC NPs maintained good stability for 48 h, with no changes in hydrodynamic size and or solution color (Figure S3 and Figure S4). UV-vis NIR absorption and emission spectra of the BTz-IC NPs were measured in tetrahydrofuran (THF) and PBS. The BTz-IC NPs exhibited a broad absorption between 600 and 900 nm, with a peak at approximately 780 nm (Figure 1D). UV-vis-NIR absorption spectra of the BTz-IC NPs in PBS solution was shown in Fig S5. Moreover, the BTz-IC NPs showed a wide emission spectrum (850-1,150 nm) when excited by an 808 nm laser (Figure 1E). In addition, NIR-II FL images gradually became brighter, and the FL signal intensity increased as the concentration of the BTz-IC NPs increased (Figure 1F and 1G).

### Therapeutic efficacy of the BTz-IC NPs in solution

**Figure 2:**
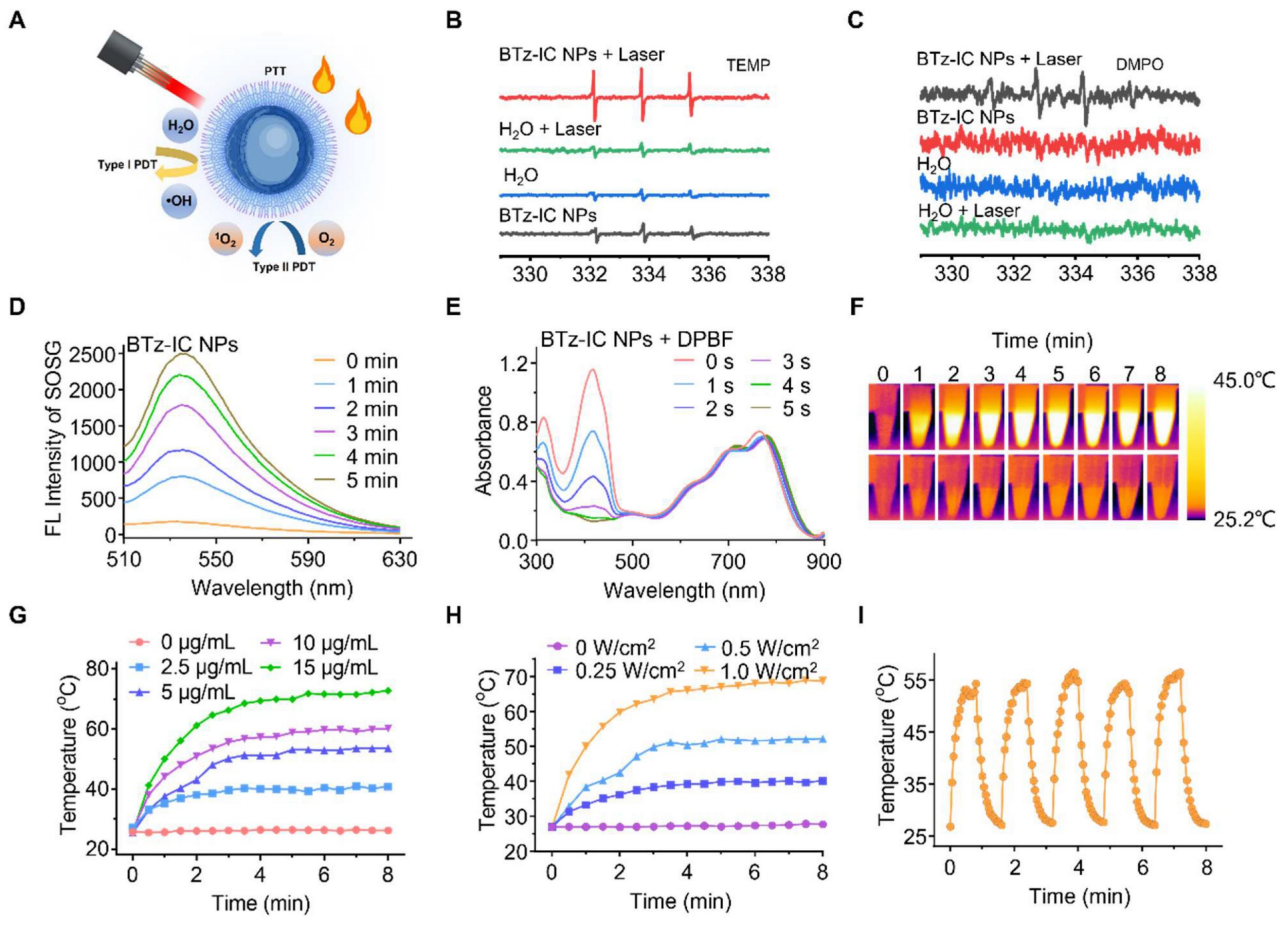
*In vitro* photodynamic, fluorescence, photothermal properties, and stability of the BTz-IC NPs. (A) Schematic illustration of the mechanism of •OH and ^1^O_2_ generation through type I and type II photodynamic procedure. (B) Detection of ^1^O_2_ analyzed by ESR measurement. (C) Detection of •OH analyzed by ESR measurement. (D) FL spectra for ^1^O_2_ using singlet oxygen sensor green (SOSG) as fluorescence probe with different irradiation time under irradiation (808 nm, 0.1 W/cm^2^) of the BTz-IC NPs. (E) ROS generation in the presence of the BTz-IC NPs in aqueous suspensions was investigated using 1,3-diphenyli-sobenzofuran (DPBF) as the ROS detector. (F) IR images of temperature changes of the BTz-IC NPs in aqueous solution (5 μg/mL) and DI water under irradiation (808 nm, 1.0 W/cm^2^) (G) Time-dependent temperature change of different concentration of the BTz-IC NPs in aqueous solutions after 808 nm laser irradiation for 8 min. (H) Temperature changes of the BTz-IC NPs (5 μg/mL) under different power laser irradiation (0, 0.25, 0.5, 1.0 W/cm^2^). (I) Temperature variation of the BTz-IC NPs with repeated 808 nm irradiation on/off for five cycles.

Next, we assessed the photodynamic/photothermal effects of the BTz-IC NPs (Figure 2A). Using DMPO and TEMP as spin traps, electron paramagnetic resonance (EPR) was used to estimate the •OH and ^1^O_2_ content and evaluate the reactive oxygen species (ROS) generation of the BTz-IC NPs under 808 nm laser irradiation. When TEMP, DMPO and the BTz-IC NPs were present, the typical resonance peak of the TEMP/^1^O_2_ adduct (typical 1:1:1 peak) and DMPO/•OH adduct (typical 1:2:2:1) under 808 nm laser irradiation indicated the type I (•OH) and type II (^1^O_2_) photodynamic effects of the BTz-IC NPs (Figure 2B and Figure 2C).

With increasing laser irradiation time, we found that the FL intensity of SOSG at 535 nm gradually increased compared with that of the control group (Figure 2D and Figure S6), indicating that the BTz-IC NPs can produce ^1^O_2_ through the type II PDT process. More importantly, 1,3-diphenylisobenzofuran (DPBF) was employed as an indicator of total ROS production. We discovered a significant decrease in the DPBF peak at 415 nm under 808 nm laser irradiation, demonstrating the robust efficacy of the BTz-IC NPs in generating ROS upon irradiation (Figure 2E and Figure S7). We verified the photodynamic effects of IR775 and the BTz-IC NPs by using DPBF as an ROS trapping agent. As shown in Figure S8, we found that the BTz-IC NPs exhibited higher ROS production under 808 nm laser irradiation.

Additionally, the BTz-IC NPs displayed good photothermal properties. Figure 2F clearly reveals that the temperature in the BTz-IC NPs + laser group reached 53.6°C within 8 min at a concentration of 5 μg/mL and a power density of 0.5 W/cm^2^, while that in the PBS + laser group showed only a mild increase to 26.2°C. When the BTz-IC NPs (0, 2.5, 5, 10, 15 μg/mL) in PBS were irradiated with an 808 nm laser (0.5 W/cm^2^) for 8 min, the temperature increased to 40.8, 53.6, 60.2 and 72.8°C, respectively (Figure 2G). Additionally, the temperature of a 5 μg/mL BTz-IC NPs solution significantly increased when irradiated at a laser power density of 0.1, 0.25, 0.5 and 1.0 W/cm^2^ (Figure 2H). The stability of the BTz-IC NPs solution under laser irradiation was recorded by monitoring the temperature variation under repeated NIR irradiation with an 808 nm laser (0.5 W/cm^2^). The results showed that the stability of the BTz-IC NPs in solution was preserved, with the irradiated agent retaining its original activity (Figure 2I). Thus, the excellent stability of the NPs under laser irradiation indicated their potential for use in repetitive and effective NIR-II FL imaging as well as PDT/photothermal therapy (PTT).

### Anti-tumor effect of the BTz-IC NPs at the cellular level

**Figure 3:**
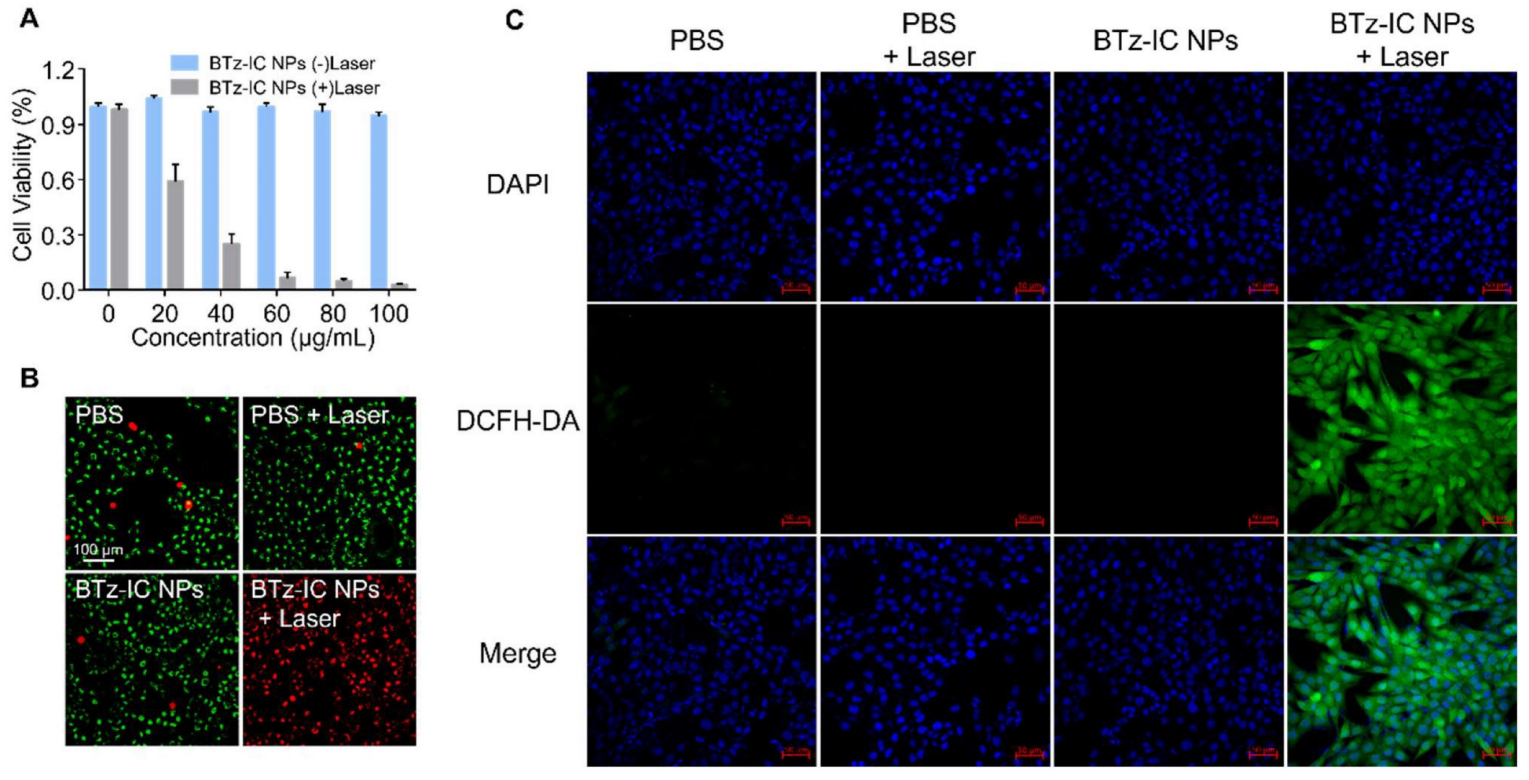
Biocompatibility, cancer therapeutic performance in Pan02 cell line. (A) MTT assay of the BTz-IC NPs in Pan02 cells at different concentrations with or without laser irradiation. (B) FL microscope imaging of Pan02 cells after PDT/PTT (scale bar = 100 μm). Viable cells are stained green with Calcein-AM, and the dead/later apoptotic cells are stained red with PI. (C) FL images of DCFH-DA in Pan02 cells excited at 488 nm laser with different treatment (first panel, DAPI; second panel, DCFH-DA fluorescence; third panel, merged images).

To explore the toxicity of BTz-IC NPs, Pan02 mouse pancreatic cancer cells were used for standard MTT analysis *in vitro*. After incubation with the BTz-IC NPs for 24 h, Pan02 cells retained more than 90% viability, even at high concentrations up to 100 μg/mL, indicating the low cytotoxicity and excellent biocompatibility of the BTz-IC NPs. In contrast, cells in the BTz-IC NPs + laser group showed significant apoptosis with increasing agent concentration. The cell viability decreased to 2.54% at a concentration of 100 μg/mL, as revealed by the MTT assay (Figure 3A). To visualize the therapeutic effects of the BTz-IC NPs *in vitro*, Pan02 cells after various treatments were stained with calcein AM/PI to determine the live/dead cell ratio. Cells treated with PBS, BTz-IC NPs, or PBS + 808 nm laser irradiation remained predominantly alive with negligible cell death. Upon treatment with the BTz-IC NPs and 808 nm laser irradiation, almost all the cells were killed (Figure 3B). To verify the effect of PDT/PTT on the viability of Pan02 cancer cells, we conducted MTT experiments with and without cooling during laser irradiation and found that the anticancer effect was caused by a combination of PDT and PTT (Figure S9). In addition, evaluation of Pan02 cell apoptosis by Annexin V-FITC/PI assay demonstrated that the combination of the BTz-IC NPs and laser irradiation induced efficient apoptosis (Figure S10). Moreover, the intracellular ROS levels in Pan02 cells were monitored using 2',7'-dichlorofluorescin diacetate (DCFH-DA) as the probe. The green FL intensity was significantly increased in the BTz-IC NPs + laser group compared with the PBS, laser-only, and BTz-IC NPs groups (Figure 3C).

### *In vivo* NIR-I/II FL imaging and cancer therapy for subcutaneous and orthotopic pancreatic tumors in mice

Due to the impressive phototherapeutic efficacy observed* in vitro*, further assessments in Pan02 tumor-bearing mice were performed. NIR-I FL imaging was first conducted in real time to monitor the BTz-IC NPs distribution and tumor-targeting ability. C57/BL6 mice with subcutaneous/orthotopic tumors were intravenously injected with BTz-IC NPs. NIR-I FL signals appeared in the tumor region, and the FL intensity gradually increased over time, suggesting the effective accumulation of the BTz-IC NPs in both subcutaneous and orthotopic tumors (Figure S11 and S12). Forty-eight hours after the intravenous injection of the BTz-IC NPs, the mice were dissected for *ex vivo* NIR-I FL imaging of major organs and tumor tissues to further assess the biodistribution of the nanoagent. The NIR-I FL intensity in the tumor tissue was stronger than that in the normal pancreas, indicating effective accumulation of the BTz-IC NPs in the tumor region (Figure S13).

**Figure 4:**
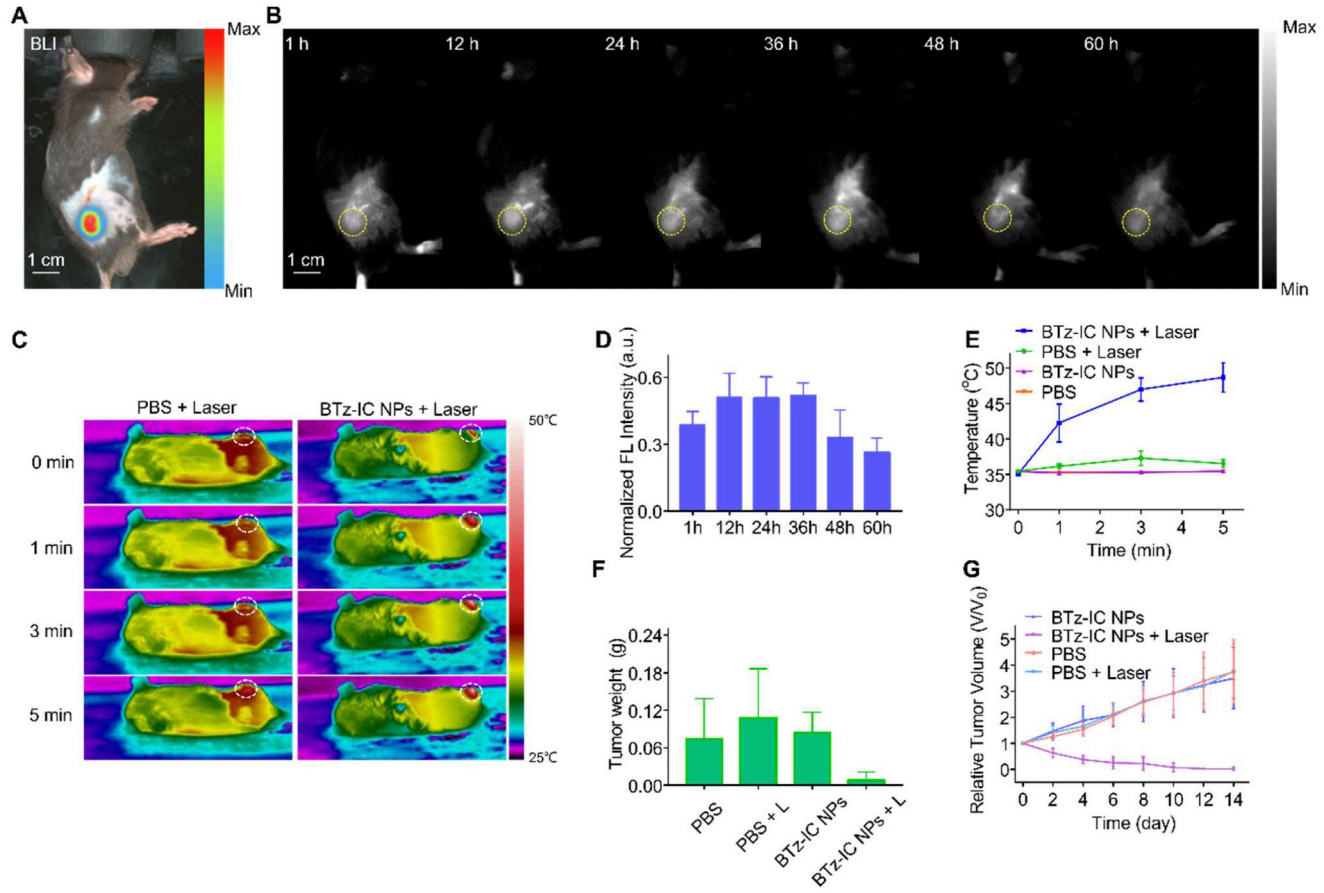
NIR-II FL images and cancer therapy of subcutaneous Pan02 tumor-bearing mice at different time points after intravenous injection of the BTz-IC NPs. (A) *In vivo* BL imaging of subcutaneous tumor. (B) NIR-II FL images of subcutaneous tumor models at various time points after intravenous injection of the BTz-IC NPs (n = 3, scale bar = 1 cm). (C) Temperature change in tumor region in each group with 808 nm laser irradiation (5 min, 1.0 W/cm^2^) after intravenous injection of PBS or the BTz-IC NPs. (D) Quantitative analysis of NIR-II FL intensity in subcutaneous tumor (n = 3). (E) Corresponding temperature variation of subcutaneous tumors in different groups. (F) Variation of tumor weights after irradiation for 14 days and (G) tumor inhibitory effect.

To estimate the *in vivo* NIR-II FL performance of the BTz-IC NPs, we further carried out NIR-II FL imaging of subcutaneous tumors and orthotopic pancreatic tumors in mice. Mice with subcutaneous tumors were injected with the BTz-IC NPs in PBS solution (1.0 mg/mL, 150 µL) and then immediately subjected to 808 nm laser excitation using a 1,040 nm filter. Subcutaneous Pan02 tumor-bearing mice were administered PBS and the BTz-IC NPs to verify the passive targeting ability of the NPs. The location of each subcutaneous tumor was determined by bioluminescence (BL) imaging (Figure 4A). NIR-II FL imaging was performed at various time points after injection, and the signal in the tumor was gradually enhanced, peaking at 12~36 h postinjection (Figure 4B and Figure 4D). Furthermore, subcutaneous tumor-bearing mice were treated with (i) PBS, (ii) BTz-IC NPs, (iii) PBS + 808 nm laser and (iv) BTz-IC NPs + laser. At 24 h after the intravenous injection of the BTz-IC NPs, mice in groups (ii) and (iv) underwent 808 nm laser irradiation (1.0 W/cm^2^, 5 min). As shown in Figure 4C and Figure 4E, upon laser irradiation, the temperature of tumors in the BTz-IC NPs + laser group increased from 35.1°C to 48.7°C in 5 min. However, the temperature of tumors in PBS + laser group only increased to 36.5°C.

An excellent antitumor effect was observed in the BTz-IC NPs + laser group compared with the other groups. As shown in Figure 4F, the tumor weight in the BTz-IC NPs + laser group decreased significantly compared with that in the other groups. However, the relative tumor volume in the BTz-IC NPs + laser group constantly decreased rapidly compared with that in the PBS, BTz-IC NPs, and PBS + laser groups (Figure 4G).

### *In vivo* NIR-II FL imaging and cancer therapy for orthotopic pancreatic tumors

**Figure 5:**
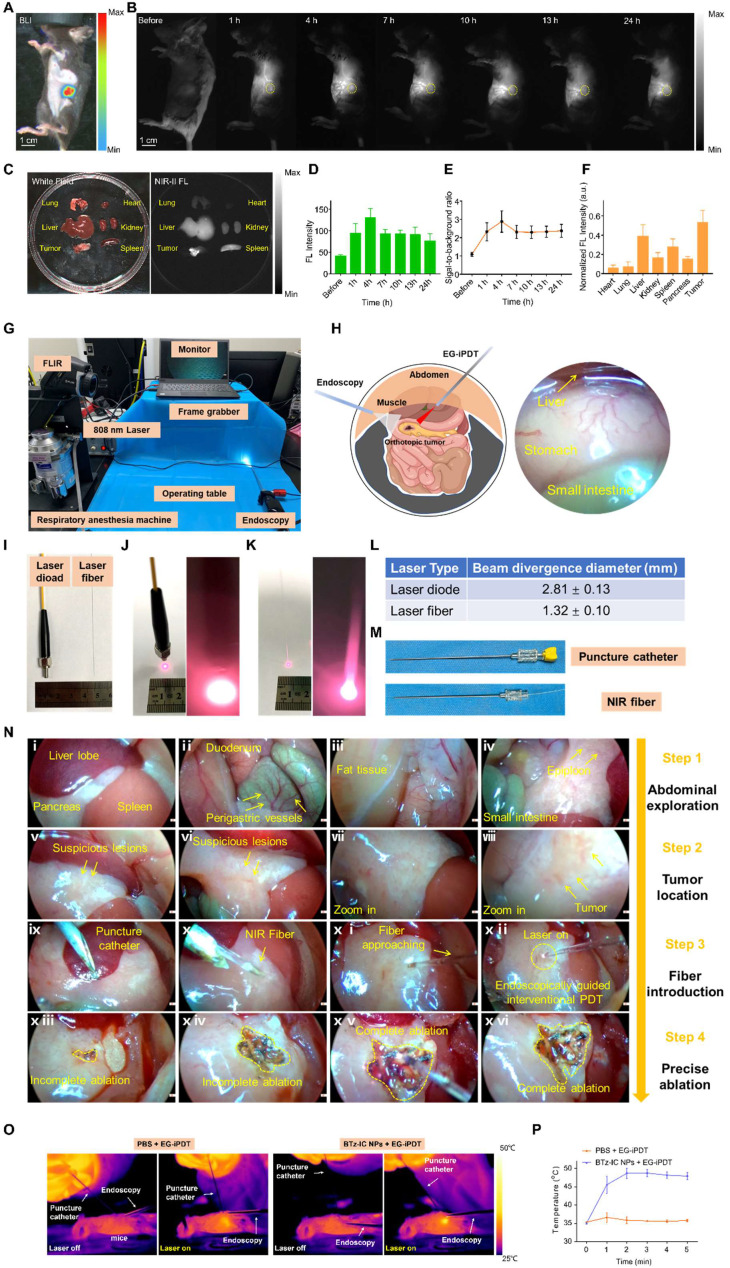
*In vivo* NIR-II FL imaging of orthotopic pancreatic tumor and self-built platform for endoscopically diagnosis and treatment of small animal. (A) *In vivo* BL imaging and (B) NIR-II FL images of orthotopic tumor models at various time points after intravenous injection of the BTz-IC NPs (n = 3, scale bar = 1 cm). (C) FL signal distribution of different organs. (D) Quantitative analysis of NIR-II FL intensity in orthotopic pancreatic tumors (n = 3) (*p<0.05). (E) Variation of signal-to-background-ratio (SBR) after intravenous injection of the BTz-IC NPs. (F) *Ex vivo* FL intensity of different organs (n = 3). Mean value and error bar are defined as mean and s.d., respectively (*p<0.05). (G) Autonomously constructed endoscopic platform for small animals. (H) Schematic illustration (left) and real images under the endoscopy (right). (I) Conventional laser diode and optical fiber (left: laser diode, right: optical fiber). (J) The spot size of the conventional laser diode after turning on the laser. (K) The spot size of the fiber (laser diode and fiber at the same height). (L) Comparison of the beam divergence diameter of conventional laser diode and optical fiber. (M) Piercing kit, piercing tube and piercing insert and NIR fiber through the puncture cannula. (N) EG-iPDT possess. (O) IR images of orthotopic tumor mice during EG-iPDT after injection of the BTz-IC NPs for 4 h. (P) *In vivo* tumor region temperature change curves of (O).

Encouraged by the excellent results in the subcutaneous tumor model, we further employed NIR-I and NIR-II FL imaging *in vivo* using the BTz-IC NPs in an orthotopic tumor mouse model. The location of the orthotopic pancreatic tumor was determined by BL imaging (Figure 5A). After the intravenous injection of the BTz-IC NPs (1 mg/mL, 125 μL) in orthotopic tumor-bearing mice, the NIR-II FL signal in the orthotopic tumor region gradually increased over time and peaked at 4 h, with a maximum signal-to-background ratio (SBR) of 2.88 (Figure 5B and Figure 5E). The NIR-II FL signal intensity in tumors was enhanced compared with that in normal pancreatic tissue, indicating the passive targeting, i.e., via the enhanced permeability and retention (EPR) effect of orthotopic pancreatic cancer.

Forty-eight hours after the intravenous injection of the BTz-IC NPs, *ex vivo* images of the major organs and tumors demonstrated that the nanoagents were mainly distributed in the liver, spleen, and tumor (Figure 5C and Figure 5F) (*p < 0.05). The FL intensity of the orthotopic tumor and in the NIR-II window was 3.55-fold higher than that of normal pancreatic tissue, demonstrating the superior imaging ability in the NIR-II window.

To construct an endoscopic treatment platform, we applied the BTz-IC NPs in EG-iPDT for orthotopic pancreatic cancer. The endoscopic imaging platform mainly consisted of imaging and ablation systems (Figure 5G). Virtual and real images of the mouse intraperitoneal cavity under endoscopy are shown in Figure 5H. A thermal imager was used to visualize the temperature change in the orthotopic pancreatic tumor-bearing mice during treatment. The diameter of the laser diode was 3.5 mm, and the diameter of the fiber was only ~0.3 mm (Figure 5I). After turning on the laser at the same height level and same power, the spot diameter of the normal laser diode was 2.81 ± 0.13 mm, while that of the fiber was only 1.32 ± 0.10 mm (Figure 5J, Figure 5K and Figure 5L). The optical fiber was inserted into the abdominal cavity through the puncture cannula (Figure 5M). Thus, it could be demonstrated that, in combination with the local magnification function of endoscopy, the fiber could converge the laser to a small region for the precise treatment of orthotopic pancreatic cancer.

Under monitoring by NIR-II FL imaging, synergistic therapeutic effects were then explored. Pan02 tumor-bearing mice were randomly divided into two groups (n = 5/group) and intravenously injected with the BTz-IC NPs (1 mg/mL, 125 µL) or PBS. The mice were anesthetized with isoflurane and placed on an operating table, and the extremities were secured with tape. The skin of the abdomen was incised to create an opening ~4 mm in diameter (see schematic diagram), and an endoscopic lens was placed.

We divided the EG-iPDT procedure for the pancreatic tumor into four steps.

Step One: Abdominal exploration was performed to check for metastases in the abdominal cavity. Endoscopy clearly showed the liver, pancreas, and spleen of the mouse (Figure 5ni), as well as the gastric fundic vessels and duodenum (Figure 5nii). The abdominal fat, small intestine, and localized abdominal adhesions (caused by primary open surgery for the injection of Pan02 tumor cells) were also observed (Figure 5niii and Figure 5niv).

Step Two: Tumor localization was performed by endoscopically searching for suspicious lesions (Figure 5nv and Figure 5nvi), clarifying the tumor region, moving the lens closer (Figure 5nvii) and determining the tumor boundary (Figure 5nviii).

Step Three: The optical fiber was introduced. Under endoscopic guidance, a puncture kit was placed (Figure 5nix), the optical fiber was introduced into the abdomen through the kit (Figure 5nx), and the laser (1.0 W/cm^2^, 5 min) was turned on for treatment (Figure 5nxi and Figure 5nxii). During treatment, ablation of the orthotopic tumor could be observed in real time by endoscopy, and lesions that were not completely ablated could be ablated again (Figure 5nxiii and Figure 5nxiv).

Step Four: Complete ablation of the tumor was achieved (Figure 5nxv and Figure 5nxvi).

During the process, photothermal images and the temperature elevation were recorded with an infrared thermal camera (Figure 5O). The tumor temperature in the BTz-IC NPs + laser group reached 49.8°C, whereas the highest temperature in the PBS + EG-iPDT group was only 38.1°C (Figure 5P).

**Figure 6:**
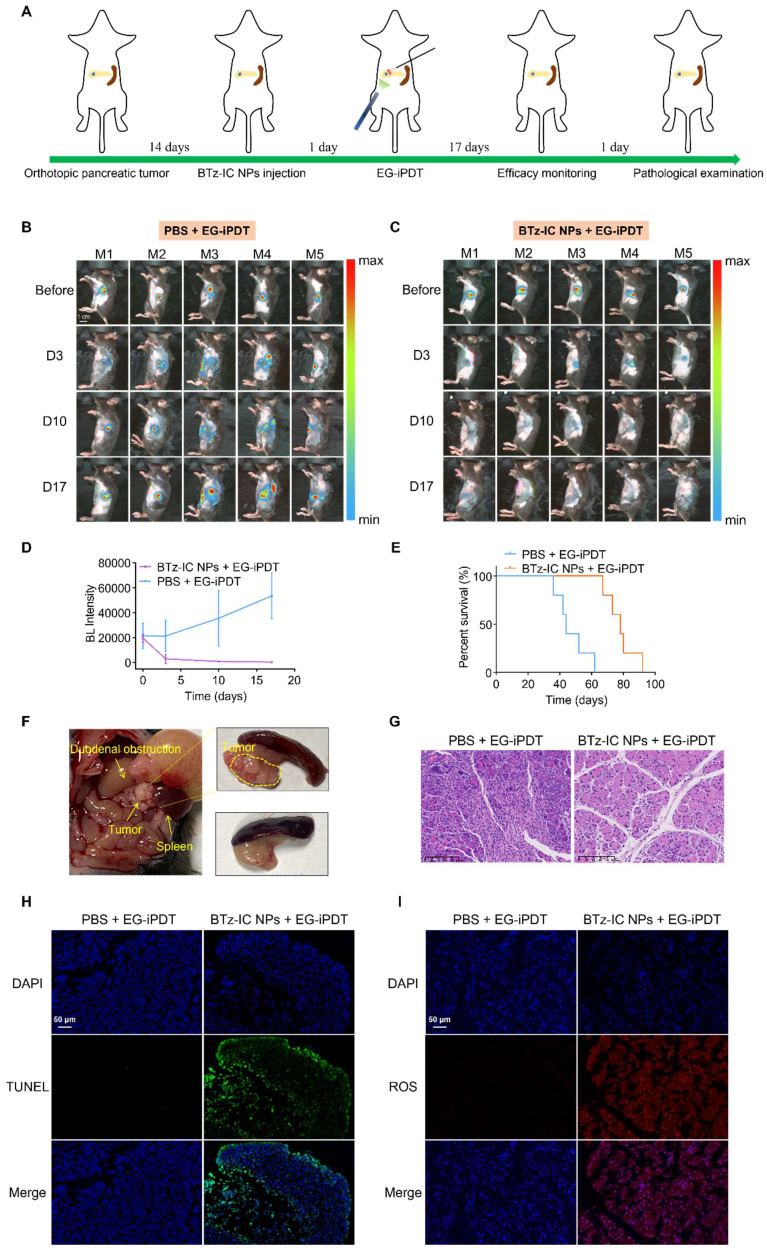
Efficacy evaluation of EG-iPDT. (A) The timeline schedule of drug administration and EG-iPDT treatment in C57/bl6 mice. (B) (C) Continuous monitoring FL signals of orthotopic pancreatic tumors by BL imaging in the PBS + EG-iPDT group (n = 5) and the BTz-IC NPs + EG-iPDT group (n = 5) for 17 days (scale bar = 1 cm). (D) Quantitative analysis of BL intensity in orthotopic pancreatic tumor-bearing mice for 17 days. (E) Survival rate of orthotopic pancreatic tumor-bearing mice after the PBS + EG-iPDT and the BTz-IC NPs + EG-iPDT. (F) Tumor grew and compressed the duodenum causing gastrointestinal obstruction in the PBS + EG-iPDT group, while no obvious tumor growth was observed in the BTz-IC NPs + EG-iPDT group. (G) H&E staining images of orthotopic tumor after different treatment (scale bar = 100 μm). (H) (I) TUNEL and ROS staining of orthotopic tumor tissue after different treatment (scale bar = 50 μm).

Intraperitoneal treatment and efficacy monitoring schedules are summarized in Figure 6A. Tumors were monitored via BL imaging on days 3, 10 and 17 after EG-iPDT. As shown in Figure 6B and Figure 6C, the BL signals in the mice treated with the BTz-IC NPs + 808 nm laser irradiation were significantly lower than those in the mice treated with PBS + laser irradiation during the observation period. Figure 6D shows that the BL intensity in the tumors was significantly lower in mice treated with the BTz-IC NPs + EG-iPDT than PBS + EG-iPDT. Survival curves showed that the treated mice had an overall survival up to ~78 days, while the untreated mice had an overall survival of only ~47.2 days (Figure 6E). In addition, without effective treatment, a series of gastrointestinal symptoms occurred as the tumor size increased. The tumors in the PBS + EG-iPDT group continued to grow, and in some mice, the tumors showed infiltrative growth, resulting in local compression of the duodenum and, in turn, gastrointestinal obstruction, while no tumor growth was observed in the BTz-IC NPs + EG-iPDT group (Figure 6F). However, in the BTz-IC+EG-iPDT group, no tumor growth was observed (Figure 6F).

To confirm apoptosis, hematoxylin and eosin (H&E)-stained tumor sections were obtained, and increased apoptosis was observed in the tumors treated with the BTz-IC NPs + laser compared with those treated with PBS + laser (Figure 6G). Terminal deoxynucleotidyl transferase dUTP nick end labeling (TUNEL) and staining for ROS were performed to further evaluate the extent of cell damage and ROS production in tumor sections after ablation. The TUNEL assay did not show significant apoptosis in the PBS + EG-iPDT group, whereas the number of apoptotic and necrotic cells was evidently increased in the BTz-IC NPs + EG-iPDT group (Figure 6H). After EG-iPDT for orthotopic tumors, intracellular ROS expression increased significantly, suggesting that the BTz-IC NPs under laser irradiation produced a significant photodynamic/photothermal antitumor effect (Figure 6I). Moreover, H&E staining of major organs was carried out to further evaluate the potential toxicity of the different treatments. No significant pathological changes were observed in the major organs in mice treated with the BTz-IC NPs (Figure S14). We also evaluated variation in the levels of inflammatory factors such as amylase (AMY), TNF-α, and IL-1β in the blood of tumor-bearing mice after different treatments. The results showed no significant changes in the BTz-IC NPs and the BTz-IC NPs + laser groups compared to the control group (Figure S15). Additionally, significant acute inflammatory changes were not observed on H&E staining in the surrounding tissues of the tumors in control, BTz-IC NPs, and BTz-IC NPs + EG-iPDT groups (Figure S16).

## Discussion

Pancreatic cancer is an invasive disease with high malignancy and a poor prognosis due to the difficulty of early detection and treatment. After more than a century of development, PDT has been clinically applied to treat various tumors and nontumor diseases, such as cancer of the skin, retina, bladder, esophagus, gastrointestinal tract, bile duct, oral cavity, head, and neck. Furthermore, PDT has attracted increasing attention as a promising complement for, or alternative to, classical surgery and interventional treatments involving drugs. This is because the photodynamic activity that exerts a tumoricidal effect occurs only upon light irradiation.

Despite the benefits of precise and effective treatment, the application of PDT is limited to surface lesions due to the insufficient penetration of light into biological tissues, preventing clinical treatment for deep abdominal tumors, particularly pancreatic tumors. However, the effect of PDT on deep abdominal tumors is weakened by the limited penetration depth of NIR light. In the relevant literature, optical fibers are mainly inserted into pancreatic tumors via US-guided percutaneous puncture, EUS, or computed tomography (CT), which may lead to bleeding or difficulty in evaluating ablation boundaries, potentially leading to failure. In addition, the limited tissue penetration depth of PDT severely hinders its applications in the treatment of deep tumors due to the difficulty of delivering sufficient irradiation energy into deep malignant tissues. There are two ways to address this challenge. The first is to increase the local power density of the NIR laser, but this will inevitably cause burns to the skin and muscles. Additionally, although long-wavelength lasers have a greater penetration depth, they are not sufficient to reach abdominal organs. The second is to use an interventional photodynamic approach in which NIR optical fibers are delivered to the tumor region for local ablation. However, there have been few reports on interventional PDT. In this work, we improved the endoscopic resolution compared to that in a previous study, enabling clearer visualization of microscopic tumors and application of the method in the treatment of orthotopic pancreatic cancer. For the treatment of deep and early-stage tumors, open surgery can be invasive, but good results can be achieved with EG-iPDT. Interventional PDT is less invasive, and complete ablation followed by fast recovery may be achieved by EG-iPDT.

However, early-stage tumors, such as those of early-stage stomach cancer and bowel cancer, can be effectively treated by endoscopic therapy, most commonly by ablative resection followed by endoscopy 15,16. Encouraged by these findings, we developed a NIR-II nanoagent based on an endoscopic platform for the early detection of orthotopic pancreatic cancer and subsequent precise PDT. We constructed a minimally invasive endoscope with a lens diameter of only 4 mm, which can be used to clearly define the location of the tumor and provide a novel strategy for the precise treatment of orthotopic pancreatic tumors by PDT. In addition, a puncture kit was utilized as a trocar for clinical laparoscopy, and an optical fiber was introduced into the abdominal cavity through the puncture kit, thus realizing EG-iPDT for orthotopic pancreatic tumors. The main advantage of this interventional method is the accurate visualization of primary and metastatic lesions in the abdominal cavity and ability to evaluate the degree of tumor ablation under direct vision.

The hypoxic TME is an intrinsic characteristic of pancreatic cancer that can reduce the efficacy of novel PDT strategies. Mounting clinical evidence has shown that the hypoxic TME in pancreatic cancer can influence both genomic stability and proteomic changes in cancer cells, thereby affecting intracellular and extracellular matrix (ECM) metabolism 17. The BTz-IC NPs were found to have a higher FL intensity and radiant efficiency than other halogen-substituted compounds along with satisfactory biocompatibility. In addition, the clinical trial and basic research of photosensitizers in pancreatic tumors was investigated (Table S1) 8,9,10,12,18-27. Inspired by the impressive *in vitro* and *in vivo* phototherapeutic performance, we discovered that the BTz-IC NPs also have excellent NIR-II FL imaging capabilities. Early-stage pancreatic tumors could be effectively detected by NIR-II FL imaging and would be suitable for type I PDT. Furthermore, we demonstrated that under 808 nm laser irradiation, not only is ^1^O_2_ generated by type II PDT through type II energy transfer, but •OH is also generated by type I PDT electron transfer. Importantly, type I PDT, which is less dependent on O_2_, can improve the efficacy of PDT for pancreatic cancer and is expected to become an alternative treatment strategy.

In the future, it is expected to develop a multifunctional endoscope with NIR-II FL imaging function for real-time guided ablation by PDT. Combining NIR-II FL imaging and endoscopy into one lens is the current trend in the development of cancer treatments. In this respect, we have conducted a simple exploration, but many technical adjustments still need to be investigated. These initial explorations have motivated us to pursue the potential clinical translation of this strategy for the treatment of early-stage pancreatic cancer that cannot feasibly be treated by radical surgery. Our findings have important implications for patient care and hold promise for clinical translation and application.

## Conclusion

In summary, we successfully developed a unique and effective endoscopically guided antitumor strategy that combines NIR-II FL with PDT for the treatment of orthotopic pancreatic cancer in mice. The developed noncytotoxic BTz-IC NPs exhibited a satisfactory NIR-II FL intensity, excellent passive targeting, and efficient ROS production. *In vivo* results confirmed that the BTz-IC NPs were able to define the boundary of subcutaneous and orthotopic tumors and overcome pancreatic tumor-associated hypoxia via the generation of •OH through type I PDT. Furthermore, we developed EG-iPDT, a minimally invasive method for ablation with a biocompatible contrast agent, to completely ablate orthotopic pancreatic tumors in a mouse model. Neither recurrence nor obvious side effects were observed during the experiments. Overall, this work proposes a potential strategy for improving the efficacy of PDT for orthotopic pancreatic cancer.

## Supplementary Material

Supplementary materials and methods, figures and table.

## Abbreviations

PDAC: pancreatic ductal adenocarcinoma
TME: hypoxic tumor microenvironment
PDT: photodynamic therapy
EUS: endoscopic ultrasonography
NIR-II: near-infrared II
FL: fluorescent
EG-iPDT: endoscopically guided interventional photodynamic therapy
AMY: amylase
