# Supplementary material for: Endoscopically guided interventional photodynamic therapy for orthotopic pancreatic ductal adenocarcinoma based on NIR-II fluorescent nanoparticles
Source: Theranostics. 2023 Aug 6;13(13):4469–81. doi: 10.7150/thno.84164 (PMC10465211; doi:10.7150/thno.84164)
Supplement: Supplementary file 1 — Supplementary materials and methods, figures and table. [file thnov13p4469s1.pdf]

Supporting information

Materials and Methods

Materials.

Chemicals and reagents were purchased from commercial sources and used without further purification. 2,7-Dichlorodihydrofluorescein diacetate (DCFH-DA) and the Calcein AM/PI Double Staining Kit were purchased from Dojindo. Singlet oxygen sensor green (SOSG) was obtained from Thermo Fisher. 5,5-Dimethyl-1-pyrroline N-oxide (DMPO) and 2,2,6,6-tetramethyl-4-piperidone (TEMP) were purchased from Aladdin. Thiazolyl blue tetrazolium bromide (MTT) was obtained from Shanghai Yuanye Science & Technology Co., Ltd. 1,2-Dimyristoyl-sn-glycero-3-phosphoethanolamine-N- [methoxy (polyethylene glycol) (DSPE-mPEG, Mw = 2000) was acquired from Shanghai ToYongBio Tech. Inc.

Characterization

Ultrapure water was produced by a Milli-Q reference system (Millipore) (18.2 MΩ/cm). Transmission electron microscopy (TEM) images of the BTz-IC NPs were recorded with a JEM-2100F transmission electron microscope (JEOL). Particle size was verified by dynamic light scattering (DLS) using a Malvern Zetasizer Nano ZS90. The absorption spectrum of the BTz-IC NPs was measured by using a UV/VIS absorption spectrometer (UV-1800, Shimadzu). Laser-induced hyperthermia was recorded with a FLIR thermal camera (FLIR Systems, Inc.). The 808 nm laser system was obtained from Changchun New Industry Laser Technology Co., Ltd. Electron-spin resonance (ESR) signals were recorded on a JES-FA 200 spectrometer at room temperature.

Synthesis of the BTz-IC NPs

The NIR-II molecule was synthesized as described in the literature[1]. Briefly, 0.3 g of 6,12,13-tris(2-ethylhexyl)-3,9-diundecyl-12,13-dihydro-6H-thieno[2'',3'':4',5']thieno [2',3':4,5]pyrrolo[3,2-g]thieno[2',3':4,5]thieno[3,2-b][1,2,3]triazolo[4,5-e]indole (SunaTech Inc.) and 1,2-dichloroethane (10 mL) were mixed in 10 mL DMF at 0°C, and 5.36 mmol of phosphorus oxychloride was added to the mixture in a nitrogen environment. Then, they were further processed and purified to obtain yellow solid compound A. Next, 0.13 mmol of compound A, 0.5 mmol of 2-(3-oxo-2,3-dihydro-1H-inden-1-ylidene) (SunaTech Inc.) malononitrile and 0.5 mmol of pyridine were mixed in 30 mL of chloroform under nitrogen and then reacted at 65°C for 20 h.

Tetrahydrofuran (THF) (1 mL) was used as a solvent to dissolve 100 μg of the BTz-IC molecules and 2.5 mg of DSPE-mPEG (2k), which were then sonicated for 1 min. Then, the THF solution was immediately added to 9 mL of water, followed by 8 min of sonication. BTz-IC @DSPE-mPEG NPs were produced after the THF was evaporated, and they were

then ultrafiltered and washed three times with water before being redispersed in 1 mL of water. Before use, BTz-IC @DSPE-mPEG was kept at 4°C. (2,2'-((2Z,2'Z)-((6,12,13-tris(2-ethylhexyl)-3,9-diundecyl-12,13-dihydro-6H-thieno[2'',3'':4',5']thieno[2',3':4,5]pyrrolo[3,2-g]thieno[2',3':4,5]thieno[3,2-b][1,2,3]triazolo[4,5-e]indole-2,10-diyl)bis(methaneylylidene))bis(3-oxo-2,3-dihydro-1H-indene-2,1-diylidene))dimalononitrile, BTz-IC)

Detection of •OH and $^1O_2$ by ESR.

To detect •OH and $^1O_2$ by ESR, DMPO and TEMP were used as spin-trapping agents. In the experiment, DMPO or TEMP was combined with the BTz-IC NPs (200 μg/mL) at a final concentration of 500 mM and irradiated with an 808 nm laser for 30 s. Within 10 min, the ESR spectra were captured. The BTz-IC NPs without laser irradiation were tested as controls.

***In vitro* photothermal experiments**

Various BTz-IC NPs concentrations in PBS were irradiated by an 808 nm laser at a power density of 0.5 W/cm^2. An infrared thermal camera captured the solution's temperature changes. Then, an 808 nm laser was used to irradiate 100 μL of PBS solution containing the same concentration of the BTz-IC NPs (5 μg/mL) at different densities (0.1, 0.25, 0.5, and 1.0 W/cm^2).

The η was calculated by equation:

$$\eta = \frac{hS(T_{Max}-T_{Sur})-Q_{Dis}}{I(1-10^{-A_{808}})} \qquad (1)$$

hS can be calculated by the follow equation:

$$hs = \frac{m_D\, C_D}{\tau_s} \qquad (2)$$

$$\theta = \frac{T-T_{sur}}{T_{max}-T_{sur}} \qquad (3)$$

$$t = -\tau_s \ln(\theta) \qquad (4)$$

h: the heat transfer coefficient

S: the surface area of the container

T_{max}: the plateau temperature (T_{max} = 44.3 °C)

T_{sur}: the surrounding temperature (T_{sur} = 26.1 °C)

Q_{dis}: the energy of solvent and the same cell under laser irradiation. (Q_{dis}= 0.001389)

I: the lase power (0.8 W/cm^2)

A_{808}: the absorbance at 660 nm (A_{808} = 0.727)

mD: the mass of solution (mD = 0.1 g)

CD: the heat capacity of water (CD = 4.2 J g-1 K-1)

According to the obtained data, the time constant (τ_s) was 81.865, the photothermal conversion efficiency of the BTz-IC NPs was calculated to be 13.1 %.

Cell experiment

For cell culture, Pan02 cells (Pan02 and Pan02-LUC cell lines were purchased from IMMOCELL (Xiamen, Fujian, China)) were cultured under 5% CO_2 at 37°C in Dulbecco's modified Eagle's medium (DMEM) (containing 1% penicillin-streptomycin (PS) and 10% fetal bovine serum (FBS)). A conventional MTT test was used to determine the cytotoxicity of the BTz-IC NPs.

For the *in vitro* PDT study, Pan02 cells (2×10^3 cells per well) were seeded on 96-well plates, cultivated for 24 h, and then mixed with various concentrations of the BTz-IC NPs (final concentration, 0, 20, 40, 60, 80, 100 μg/mL). After 6 h of incubation, the cells were gently washed three times with PBS to remove noninternalized nano-agents. The cells were then exposed to 808 nm laser irradiation (0.8 W/cm^2) for 5 min before being cultivated for another 12 h. To verify photodynamic and photothermal therapy, the irradiation process (0.5 W/cm^2,5 min) was completed on ice. Finally, the MTT test was used to measure cell viability. The solution was removed carefully, and MTT formazan was dissolved by adding dimethyl sulfoxide (DMSO). The absorbance of each sample solution was measured by using a microplate reader at 490 nm to determine cell viability.

Live/dead cell analysis (calcein AM/propidium iodide) was performed to evaluate the *in vitro* PDT activity of the BTz-IC NPs. Pan02 cells seeded in six-well plates were treated with the BTz-IC NPs (50 μg/mL) or PBS, without or with 808 nm laser irradiation ((i) PBS, (ii) PBS + Laser, (iii) BTz-IC NPs and (iv) BTz-IC NPs + Laser). The resulting cells were stained with calcein-AM/PI and observed by confocal laser scanning microscopy (CLSM) (Olympus FV1200).

Evaluated cell apoptosis through flow cytometry. Inoculated Pan02 cells into 12-well plates (1 per well × 10^5 cells). Co staining strategy of Annexin V-FITC and PI (Multi Sciences) cell co-staining for evaluating the apoptosis rate of Pan02 cells after different treatments ((i) PBS, (ii) PBS + Laser, (iii) BTz-IC NPs and (iv) BTz-IC NPs + laser).

Intracellular ROS levels were determined using the oxidation-sensitive fluorescent dye DCFH-DA. Pan02 cells were washed twice with PBS and incubated with 50 μg/mL BTz-IC NPs or PBS for 4 h, followed by incubation with DCFH-DA (10 μM) and Hoechst 33342 (10 μg/mL) for 30 min each. Later, the cells were washed with PBS buffer three times. The cells were then irradiated with an NIR laser (808 nm) at a power density of 0.8 W/cm^2 for 5 min and then imaged again ((i) PBS. (ii) PBS + laser, (iii) BTz-IC NPs and (iv) BTz-IC NPs + laser). The FL of DCFH-DA was observed at 485 nm excitation and 535 nm emission by CLSM.

Animal Experiment.

Animal model C57/BL6 mice were obtained from Hunan SJA Laboratory Animal Co., Ltd. All animal experiments were approved by the Institutional Animal Care and Use Committee of Hunan Provincial Hospital (No.202177). For the Pan02 tumor model, 1×10^6 Pan02 fluc cells were subcutaneously injected into the right backs of C57/BL6 mice. For the orthotopic pancreatic tumor model, Pan02 fluc cancer cells (5×10^5) were injected directly into the tail of the pancreas, and establishment of the orthotopic pancreatic tumor model was confirmed by bioluminescence imaging (BLI) as previously reported (intraperitoneal injection, 3.75 μL/g, 15 mg/mL). Tumor sizes were calculated using the following formula: volume = (length × width^2)/2.

NIR-I FL imaging. For *in vivo* NIR-I FL imaging, the mice were anesthetized using 2% isoflurane and fixed with tape. Adjust the imaging wavelength of the small animal live imaging system (VILBER FUSION-FX7) to 780 nm. After injecting BTz-IC NPs through the tail vein (orthotopic tumor: 1 mg/mL, 125 μL, subcutaneous tumor: 1 mg/mL, 150 μL), images are taken at the designated time point after injection.

NIR-II FL imaging. The BTz-IC NPs were dissolved in PBS at different concentrations and placed in the holders. The NIR-II signal intensity was measured with a near-infrared InGaAs area array imaging detector (NIRvana 640, America). For *in vivo* NIR-II FL imaging (filters: 1,040 nm, laser power density:1.60 W, exposure time: 500 ms), the mice were anesthetized using 2% isoflurane and fixed with tape. They were then irradiated with the 808 nm laser after the injection of the BTz-IC NPs (orthotopic tumor: 1 mg/mL, 125 μL, subcutaneous tumor: 1 mg/mL, 150 μL), and images were taken at designated time points after injection. The NIR-II signal intensity was analyzed by using ImageJ software.

***In vivo* cancer therapy of subcutaneous tumors.** For subcutaneous tumors, the tumor-bearing mice were randomly divided into four groups and received the following treatments: (i) PBS injection, (ii) PBS + 808 nm laser, (iii) BTz-IC NPs and (iv) BTz-IC NPs + 808 nm laser. To evaluate the *in vivo* therapy effects, the mice received intravenous administration of PBS or BTz-IC NPs (1 mg/mL, 150 μL). Twelve hours after injection of the BTz-IC NPs or PBS the mice were anesthetized with 2% isoflurane, and the subcutaneous tumors were exposed to the optical fiber and subsequently irradiated by an 808 nm laser (1.0 W/cm^2, 5 min). The tumor volume was measured every 2 days. Fourteen days after PDT, the subcutaneous tumors of mice in each group were dissected, and the tumor weights were calculated.

***In vivo* endoscopically guided interventional cancer therapy.** Orthotopic pancreatic tumor-bearing mice were randomly divided into two groups: (i) BTz-IC NPs + EG-iPDT group and (ii) PBS + EG-iPDT group. After 4 h post injection (BTz-IC NPs: 1 mg/mL,125

μL, PBS: 125 μL), the mice were anesthetized with isoflurane (2%) and placed on the operating table with the extremities secured with tape. The imaging system was composed of a small animal laparoscope with a lens diameter of 4 mm, a length of 175 mm, a field of view of 75 degrees, an angle of view of 30 degrees, and a camera resolution of 1,920 × 1,080. The fiber optics were specifically customized, with one end of the fiber connected to an 808 nm laser through the SMA-905 interface, the other end through a puncture kit entered the abdomen to directly irradiate the tumor site. A piece of skin approximately 4 mm in diameter was cut on the left abdominal wall, and an endoscopic lens was placed. We divided the endoscopically guided orthotopic tumor into four steps. (1) Abdominal exploration to check for metastases in the abdominal cavity. (2) Tumor localization, endoscopically searching for suspicious lesions, clarifying the tumor region, pulling the lens closer and determining the tumor boundary. (3) Introduction of an optical fiber. (4) Complete ablation of the tumor was achieved. Optical excitation was provided by an 808 nm diode laser coupled to an optical fiber. The BTz-IC NPs + EG-iPDT treated groups were irradiated with an 808 nm laser (1.0 W/cm^2, 5 min) at 24 h post-injection. The temperature variation of the tumor region was recorded with an infrared thermal camera (FLIR Systems Inc). After treatment, bioluminescence imaging (BLI) was used to continuously monitor the pancreatic orthotopic tumor at day 3, day 10 and day 17 (VILBER FUSION) (intraperitoneal injection, 3.75 μL/g, 15 mg/mL). Furthermore, the subcutaneous, orthotopic tumors and major organs were collected and stained with hematoxylin and eosin (H&E), FITC-conjugated terminal deoxynucleotidyl transferase-mediated dUTP nick end labeling (TUNEL) and reactive oxygen species (ROS) staining.

Statistical analysis

Data are presented as the mean ± SD and were compared by Student's t test. Statistical analysis was performed by GraphPad Prism 7.0 software and $p < 0.05$ was considered statistically significant.

Photosensitizer	Laser	Object	Imaging Guidance	Type of PDT	Tumor type	Reference
BTz-IC NPs	808 nm	mouse	Endoscopy	I/II	Orthotopic tumor	Our work
mTHPC	652 nm	Patient Phase I	US	II	Orthotopic tumor	[2]
Verteporfin	690 nm	Patient phase I/II	CT	II	Orthotopic tumor	[3]
Photolon	660 nm	Patient Phase I	EUS	II	Orthotopic tumor	[4]

Photofrin	630nm	Patient Phase I	EUS	II	Orthotopic tumor	[5]
Verteporfin	50 J/cm	Patient Phase I	EUS	II	Orthotopic tumor	[6]
DCTBT-loaded liposomes	808 nm	mouse	Endoscopy	I	Orthotopic tumor	[7]
TPMIL	690 nm	mouse	Laparotomy	II	Orthotopic tumor	[8]
PSPP-Au -D	680 nm	mouse	Laparotomy	II	Orthotopic tumor	[9]
EGaPs	690 nm	mouse	Laparotomy	II	Subcutaneous tumor	[10]
Ce6-MMAE-KGDEVD	532 nm	mouse	Laparotomy	II	Orthotopic tumor	[11]
PF11DG	655 nm	mouse	Laparotomy	II	Subcutaneous tumor	[12]
Chlorin e6-loaded R-Exo	671 nm	mouse	Laparotomy	II	Subcutaneous tumor	[13]
HCJSP prodrug nanoparticle	671 nm	mouse	Laparotomy	II	Subcutaneous tumor	[14]
MSNP5	685 nm	mouse	Laparotomy	II	Subcutaneous tumor	[15]

Table S1 Photosensitizers used in clinical and basic research of pancreatic cancer. US, ultrasound; CT, computed tomography; EUS, endoscopic ultrasound

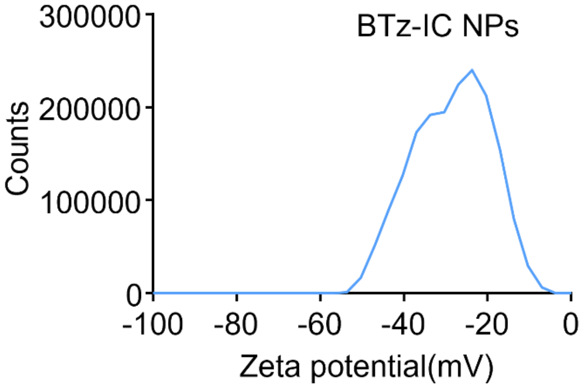

Figure S1 Zeta potential of the BTz-IC NPs.

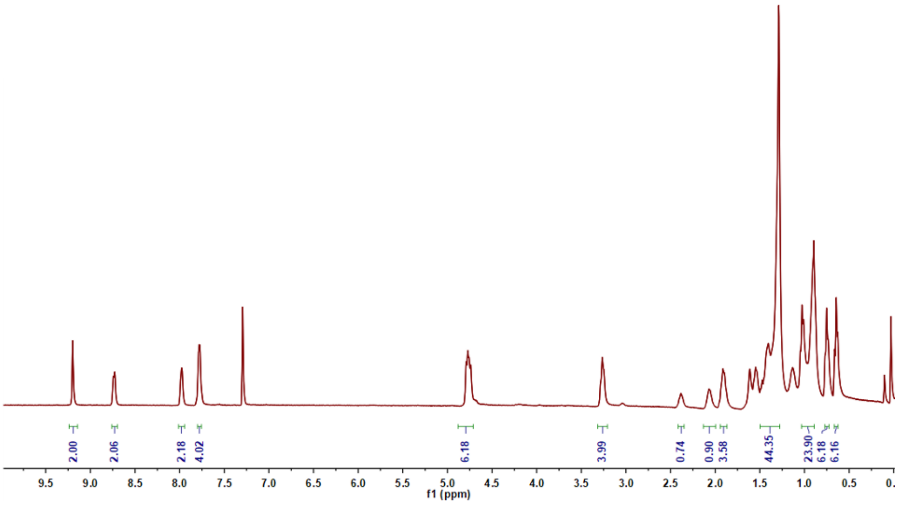

Figure S2 ^{1}H NMR spectrum of the BTz-IC.

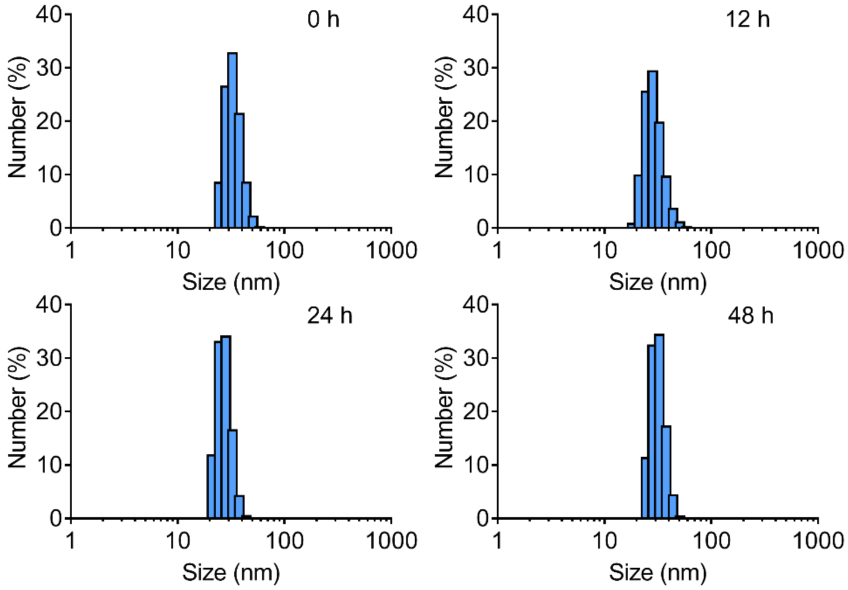

Figure S3 Size distribution of the BTz-IC NPs at different time points (0 h, 12 h, 24 h and 48 h).

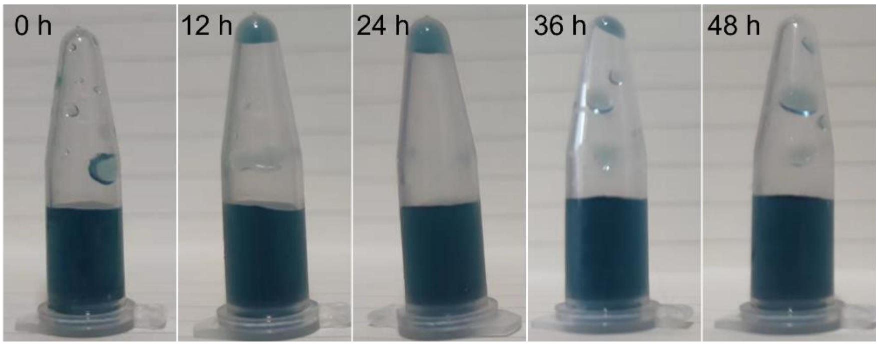

Figure S4 Photos of the BTz-IC NPs at different times (0, 12, 24, 36, 48 h).

.

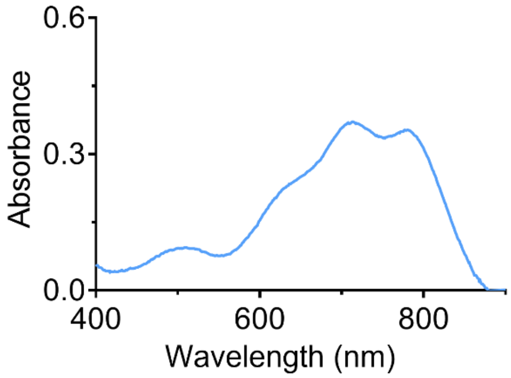

Figure S5 UV-vis-NIR absorption spectra of the BTz-IC NPs in PBS solution.

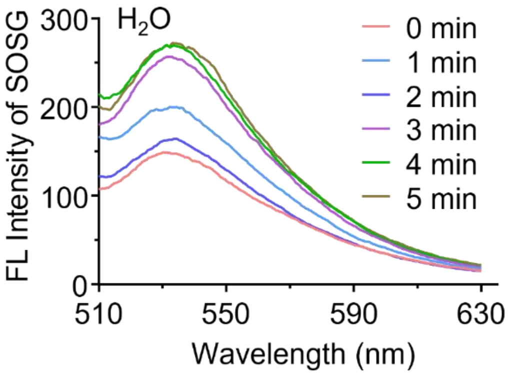

Figure S6 FL spectra for singlet-oxygen generation ($^1O_2$) using singlet oxygen sensor green (SOSG) as fluorescence probe with different irradiation time under laser irradiation (808 nm, 0.1 W/cm^2) of H_2O.

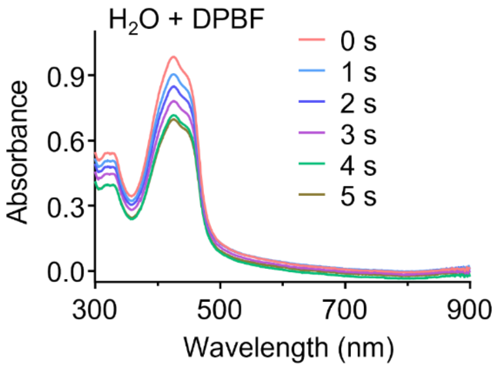

Figure S7 ROS generation in the presence of H_2O was investigated using 1,3-diphenylisobenzofuran (DPBF).

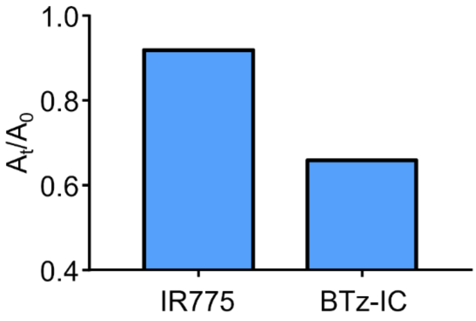

Figure S8 The absorption ratio of ROS trapping agent DPBF at 415 nm for IR775 and BTz-IC NPs with the same absorption at 808 nm before and after the same laser irradiation (A_t: the absorption value at 415 nm after 30 s of 808 nm laser irradiation, A_0: the absorption value at 415 nm before 808 nm laser irradiation (0.2 W/cm^2)).

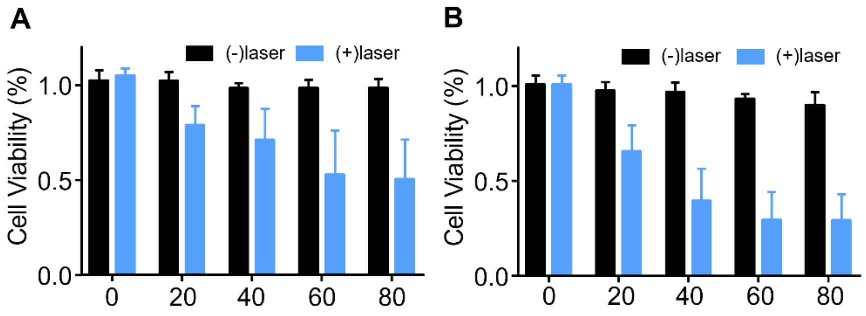

Figure S9 MTT assay of the BTz-IC NPs in Pan02 cells at different concentrations with or without laser irradiation. (a) Pan02 cells on ice during laser irradiation, to remove the photothermal effect. (b) Pan02 cells in normal temperature during laser irradiation.

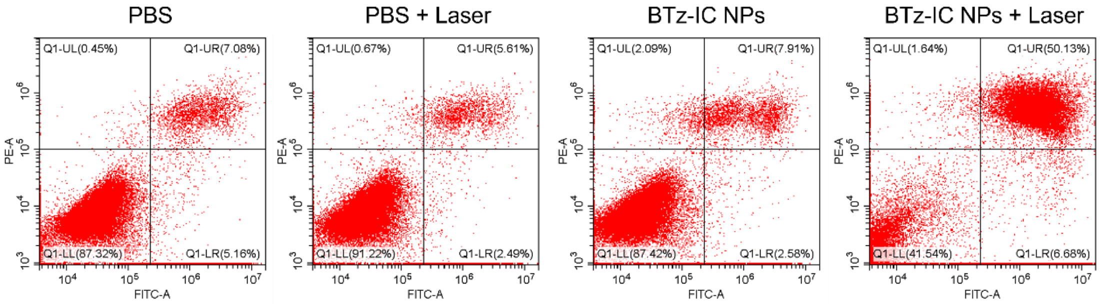

Figure S10 Flow cytometry detection of Pan02 cells incubated with PBS or the BTz-IC NPs for 24 h with/without laser irradiation (808 nm, 0.8 W/cm^2, 5 min).

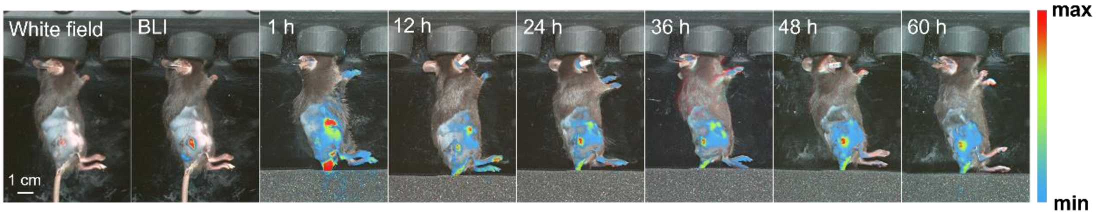

Figure S11 NIR-I imaging of Pan02 subcutaneous tumor at different time points after intravenous injection of the BTz-IC NPs (scale bar = 1 cm).

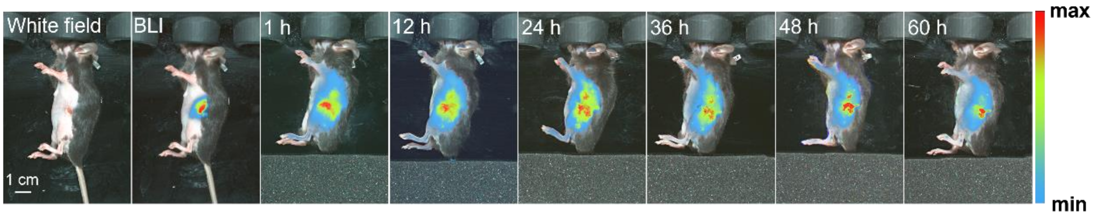

Figure S12 *In vivo* NIR-I FL images of orthotopic pancreatic tumor bearing mice after tail vein injection of the BTz-IC NPs at different time points (scale bar = 1 cm).

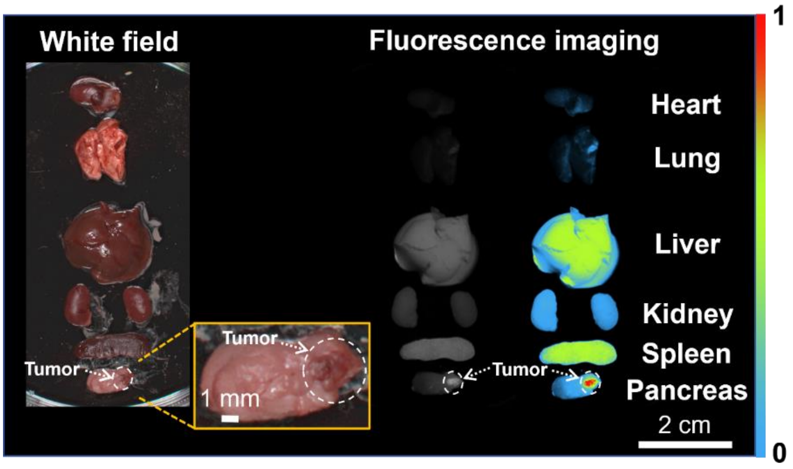

Figure S13 *Ex vivo* NIR-I FL images of tumor and major organs after 24 h i.v. injection of the BTz-IC NPs.

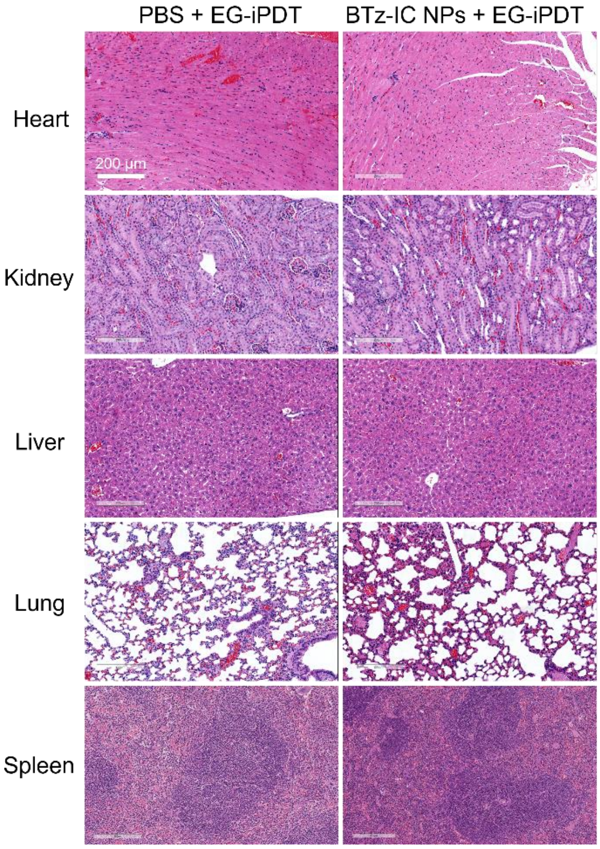

Figure S14 H&E staining of the heart, kidney, liver, lung, and spleen in mice harvested from the PBS + EG-iPDT and the BTz-IC NPs + EG-iPDT group (scale bar = 200 μm).

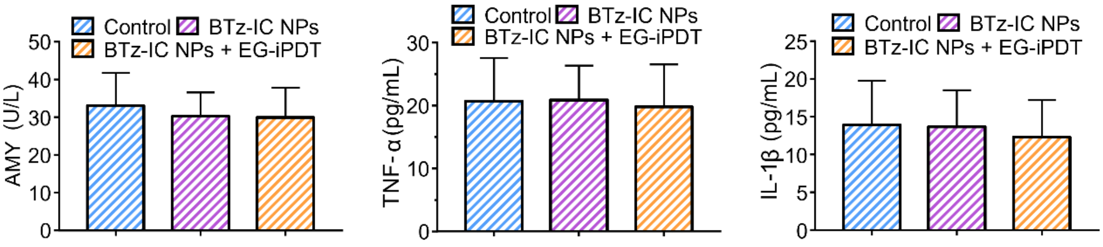

Figure S15 AMY, TNF-α, IL-1β of orthotopic tumor-bearing mice in the control, BTz-IC NPs, and BTz-IC NPs + EG-iPDT group.

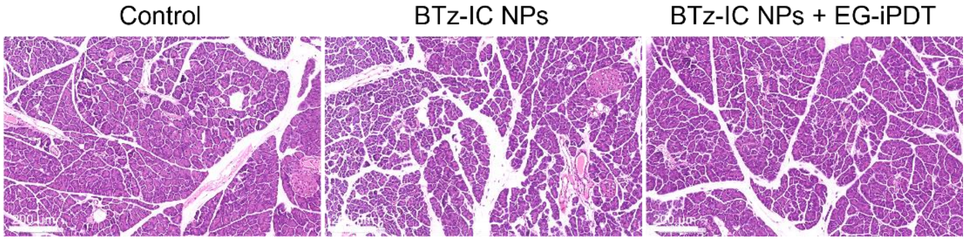

Figure S16 HE stanning images of the surrounding tissues around the tumors for control, BTz-IC NPs, and BTz-IC NPs + EG-iPDT groups.

Reference

1. Yin B, Qin Q, Li Z, Wang Y, Liu X, Liu Y, et al. Tongue cancer tailored photosensitizers for NIR-II fluorescence imaging guided precise treatment. Nano Today. 2022; 45: 101550.
2. Bown SG, Rogowska AZ, Whitelaw DE, Lees WR, Lovat LB, Ripley P, et al. Photodynamic therapy for cancer of the pancreas. Gut. 2002; 50: 549-57.
3. Huggett MT, Jermyn M, Gillams A, Illing R, Mosse S, Novelli M, et al. Phase I/II study of verteporfin photodynamic therapy in locally advanced pancreatic cancer. Br J Cancer. 2014; 110: 1698-704.
4. Choi JH, Oh D, Lee JH, Park JH, Kim KP, Lee SS, et al. Initial human experience of endoscopic ultrasound-guided photodynamic therapy with a novel photosensitizer and a flexible laser-light catheter. Endoscopy. 2015; 47: 1035-8.
5. DeWitt JM, Sandrasegaran K, O'Neil B, House MG, Zyromski NJ, Sehdev A, et al. Phase 1 study of EUS-guided photodynamic therapy for locally advanced pancreatic cancer. Gastrointest Endosc. 2019; 89: 390-8.
6. Hanada Y, Pereira SP, Pogue B, Maytin EV, Hasan T, Linn B, et al. EUS-guided verteporfin photodynamic therapy for pancreatic cancer. Gastrointest Endosc. 2021; 94: 179-86.
7. Li D, Chen X, Wang D, Wu H, Wen H, Wang L, et al. Synchronously boosting type-I photodynamic and photothermal efficacies via molecular manipulation for pancreatic cancer theranostics in the NIR-II window. Biomaterials. 2022; 283: 121476.
8. Obaid G, Bano S, Thomsen H, Callaghan S, Shah N, Swain JWR, et al. Remediating Desmoplasia with EGFR-Targeted Photoactivable Multi-Inhibitor Liposomes Doubles Overall Survival in Pancreatic Cancer. Adv Sci (Weinh). 2022; 9: e2104594.
9. Zhang S, Li Z, Wang Q, Liu Q, Yuan W, Feng W, et al. An NIR-II Photothermally Triggered "Oxygen Bomb" for Hypoxic Tumor Programmed Cascade Therapy. Adv Mater. 2022; 34: e2201978.
10. Hafiz SS, Xavierselvan M, Gokalp S, Labadini D, Barros S, Duong J, et al. Eutectic Gallium-Indium Nanoparticles for Photodynamic Therapy of Pancreatic Cancer. ACS Appl Nano Mater. 2022; 5: 6125-39.
11. Cho IK, Shim MK, Um W, Kim JH, Kim K. Light-Activated Monomethyl Auristatin E Prodrug Nanoparticles for Combinational Photo-Chemotherapy of Pancreatic Cancer. Molecules. 2022; 27.
12. Wang Z, Gong X, Li J, Wang H, Xu X, Li Y, et al. Oxygen-Delivering Polyfluorocarbon Nanovehicles Improve Tumor Oxygenation and Potentiate Photodynamic-Mediated Antitumor Immunity. ACS Nano. 2021; 15: 5405-19.
13. Jang Y, Kim H, Yoon S, Lee H, Hwang J, Jung J, et al. Exosome-based photoacoustic imaging guided photodynamic and immunotherapy for the treatment of pancreatic cancer. J Control Release. 2021; 330: 293-304.
14. Sun F, Zhu Q, Li T, Saeed M, Xu Z, Zhong F, et al. Regulating Glucose Metabolism with Prodrug Nanoparticles for Promoting Photoimmunotherapy of Pancreatic Cancer. Adv Sci (Weinh). 2021; 8: 2002746.
15. Er O, Tuncel A, Ocakoglu K, Ince M, Kolatan EH, Yilmaz O, et al. Radiolabeling, In

Vitro Cell Uptake, and In Vivo Photodynamic Therapy Potential of Targeted Mesoporous Silica Nanoparticles Containing Zinc Phthalocyanine. Mol Pharm. 2020; 17: 2648-59.
